# 15-year survivorship analysis of an interspinous device in surgery for single-level lumbar disc herniation

**DOI:** 10.1186/s12891-021-04929-8

**Published:** 2021-12-09

**Authors:** Yoon Joo Cho, Jong-Beom Park, Dong-Gune Chang, Hong Jin Kim

**Affiliations:** 1 Department of Orthopaedic Surgery, Uijeongbu St. Mary’s Hospital, College of Medicine, The Catholic University of Korea, 271 Cheonbo-ro, Uijeongbu-si, Seoul, Gyeonggi-do 11765 South Korea; 2 Department of Orthopaedic Surgery, Sanggye Paik Hospital, College of Medicine, Inje University, Seoul, South Korea

**Keywords:** Survivorship, DIAM, Lumbar disc herniation, Reoperation, Risk factors

## Abstract

**Background:**

Interspinous devices have been introduced as alternatives to decompression or fusion in surgery for degenerative lumbar diseases. This study aimed to investigate 15-year survivorship and risk factors for reoperation of a Device for Intervertebral Assisted Motion (DIAM) in surgery for 1-level lumbar disc herniation (LDH).

**Methods:**

A total of 94 patients (54 men and 40 women) underwent discectomy and DIAM implantation for 1-level LDH, with a mean follow-up of 12.9 years (range, 6.3–15.3 years). The mean age was 46.2 years (range, 21–65 years). Sixty-two patients underwent DIAM implantation for L4–5, 27 for L5–6, and 5 for L3–4. Reoperations due to any reason associated with DIAM implantation level or adjacent levels were defined as failure and used as the end point of determining survivorship.

**Results:**

During the 15-year follow-up, 8 patients (4 men and 4 women) underwent reoperation due to recurrence of LDH at the DIAM implantation level, a reoperation rate of 8.5%. The mean time to reoperation was 6.5 years (range, 0.8–13.9 years). Kaplan-Meier analysis showed a cumulative survival rate of the DIAM implantation of 97% at 5 years, 93% at 10 years, and 92% at 15 years after surgery; the cumulative reoperation rate of the DIAM implantation was 3% at 5 years, 7% at 10 years, and 8% at 15 years after surgery. Mean survival time was predicted to be 14.5 years (95% CI, 13.97–15.07). The log-rank test and Cox proportional hazard model showed that age, sex, and location did not significantly affect the reoperation rate of DIAM implantation.

**Conclusions:**

Our results showed that DIAM implantation significantly decreased reoperation rate for LDH in the 15-year survivorship analysis. We suggest that DIAM implantation could be considered a useful intermediate step procedure for LDH surgery. To the best of our knowledge, this is the longest follow-up study in which surgical outcomes of interspinous device surgery were reported.

## Background

LDH is the most common cause of surgery for degenerative lumbar diseases. Despite the development of various surgical techniques for LDH, postoperative recurrence is an unavoidable complication and often requires reoperation [1–3]. The reoperation rate can vary greatly depending on number of patients included in the study, surgical techniques, and follow-up duration and dropout [4–14]. Therefore, results of studies using s large-scale nationwide database and survival analysis are more meaningful than results of a single clinical study and have the advantages of reducing variability and errors [15]. According to a nationwide cohort study by Kim et al., reoperation rates at 5 years were 13.8 and 12.4% after open discectomy and endoscopic discectomy, respectively [14]. Retrospective analysis of USA national insurance billing database showed overall 4-year reoperation rate of 12.2% after 1-level discectomy [10]. Survival analysis for lumbar discectomy on a national scale showed the rate of revision discectomy of 7% up to 7 years for patients undergoing primary discectomy alone [15]. The results of these studies showed that patients who underwent lumbar disc surgery had a high reoperation rate due to recurrence, indicating additional measures to reduce recurrence are needed when lumbar discectomy is performed.

Recurrent lumbar disc herniation (LDH) is one of the most common lumbar discectomy-related complications [4–8]. Recurrent LDH is the presence of herniated disc material at the same level in a patient who has experienced a pain-free interval of at least 6 months after surgery on the same disc level [9–14]. Recurrent LDH can be managed with aggressive conservative treatments, but some patients require reoperation [1–3]. The reported reoperation rate after lumbar discectomy is 6–25% [4–14]. Reoperation after lumbar discectomy is a significant burden for patients and spine surgeons due to less satisfactory surgical outcomes, high hospital costs, and complications compared with primary lumbar discectomy. Therefore, prevention of reoperation is an important goal for patients and spine surgeons.

The Device for Intervertebral Assisted Motion (DIAM) is a relatively new interspinous device and restricts segmental motion, such as extension and flexion, but allows full range of axial rotation and lateral bending at the level of implantation [16–20]. Therefore, the DIAM does not increase motion and intradiscal pressure at the adjacent levels, which theoretically can prevent the development of adjacent segment pathologies. After Taylor et al. first reported the surgical technique and initial results of the DIAM in 2007, favorable surgical outcomes of short- and mid-term follow-up have been reported [21–23]. In addition, promising results of biomechanical testing for DIAM as a concept of dynamic stabilization have been reported [15, 24]. However, detailed biomechanical testing to predict DIAM survival for at least 10 years of in vivo use has not been performed. Therefore, we believe previous studies do not adequately show the actual long-term effects of DIAM in surgery for degenerative lumbar diseases. Therefore, in the present study, the 15-year survivorship analysis of DIAM surgery for LDH, which is the longest long-term follow-up period to date, was performed and results reported.

## Methods

Between January 2006 and December 2015, 313 consecutive patients underwent decompressive surgery, such as discectomy or laminectomy, and DIAM implantation for 1-, 2-, or 3-level degenerative lumbar disease. Inclusion criteria in the current study were 1-level discectomy and DIAM implantation for LDH and minimum 5-year follow-up after surgery. Exclusion criteria in the current study were 1) ≥2-level discectomy and DIAM implantation for LDH; 2) laminectomy and DIAM implantation for lumbar spinal stenosis or degenerative spondylolisthesis; 3) any history of previous lumbar spine surgeries; and 4) any history of infection or tumor of the lumbar spine. Among 313 patients, 94 met the inclusion criteria and were included in this study; 54 were men and 40 were women, with a mean age of 46.2 years (range, 21–65 years). The mean follow-up after surgery was 12.9 years (range, 6.3–15.3 years). Sixty-two patients underwent 1-level discectomy and DIAM implantation for L4–5, 27 for L5–6, and 5 for L3–4. As described in a previous paper [18], 27 patients who underwent DIAM implantation for L5–6 had a lumbosacral transitional vertebra. Therefore, S1 is lumbarized to become L6, which has a prominent spinous process so that DIAM can be implanted at L5–6.

The survivorship analysis in the current study was the same as described in a previous paper [19]. Reoperation due to any reason at the DIAM level or adjacent levels was defined as failure and used as the end point of determining survivorship. Survival time for analysis of the patients with DIAM implantation was calculated from the date of primary surgery to the date of reoperation, loss of follow-up, or December 31, 2015, whichever came first. The cumulative reoperation rate and survival time were determined using Kaplan-Meier analysis. The variables for univariate and multivariate analyses were age (< 50 years vs. ≥50 years), sex (man vs. woman), and location of DIAM implantation (L4–5 vs. L5–6 vs. L3–4). Multiple stepwise regression with Cox proportional hazard model was used to investigate the risk factors for reoperation of DIAM implantation. In addition, chi-square test of independence for parametric data and Fisher’s exact test for non-parametric data were performed. A *p*-value < 0.05 was considered as statistically significant.

## Results

**Fig. 1:**
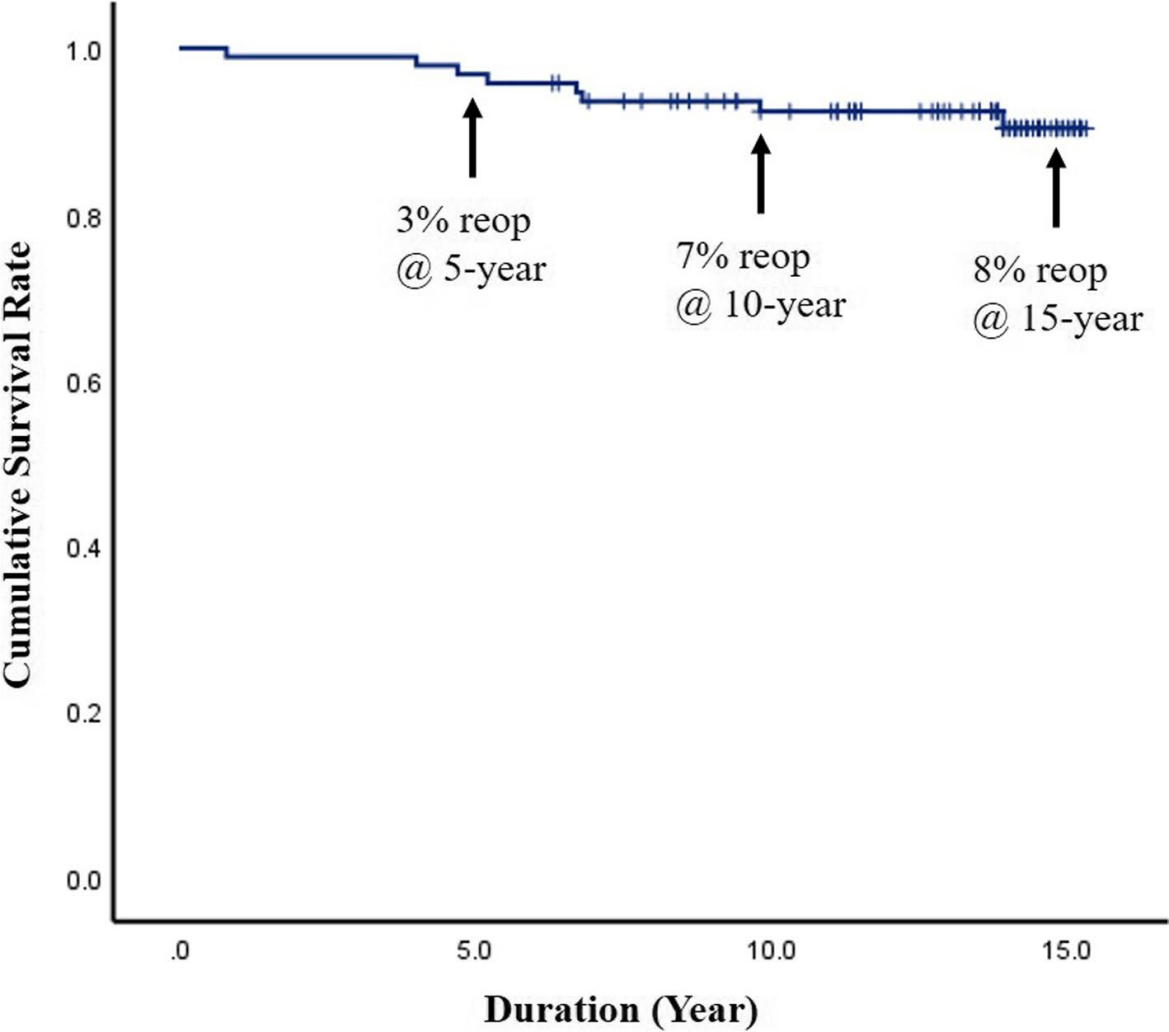
Kaplan–Meier analysis showed the cumulative survival rate of DIAM implantation in surgery for 1-level lumbar disc herniation to be 99% at 1 year, 97% at 5 years, 93% at 10 years, and 92% at 15 years after surgery; the cumulative reoperation rate of DIAM implantation was 1% at 1 year, 3% at 5 years, 7% at 10 years, and 8% at 15 years after surgery

**Fig. 2:**
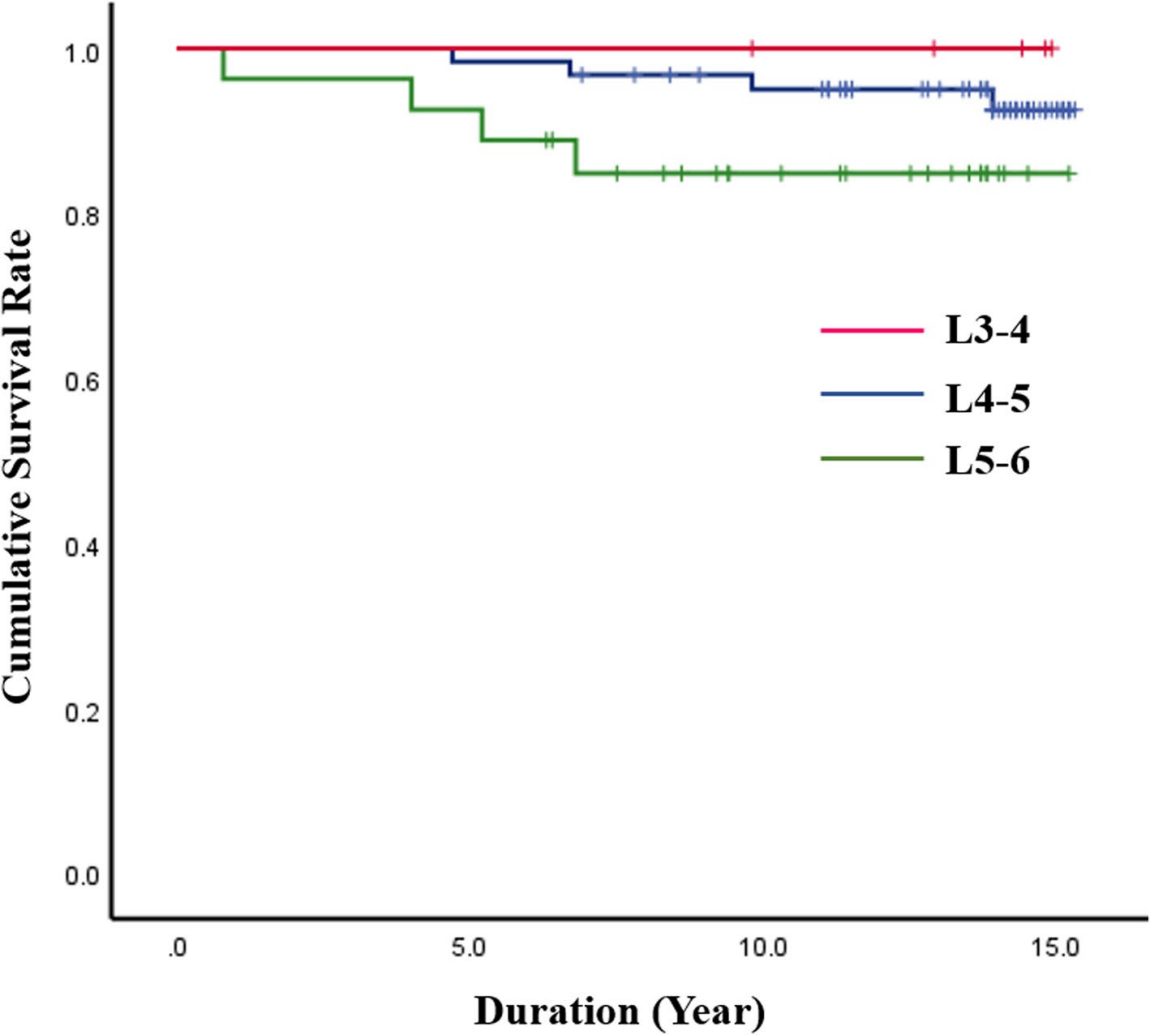
Log-rank test showed that location did not significantly affect the reoperation rate of DIAM implantation in surgery for 1-level lumbar disc herniation (L4–5 7.5% vs. L5–6 15.2%, *p* = 0.191)

**Fig. 3:**
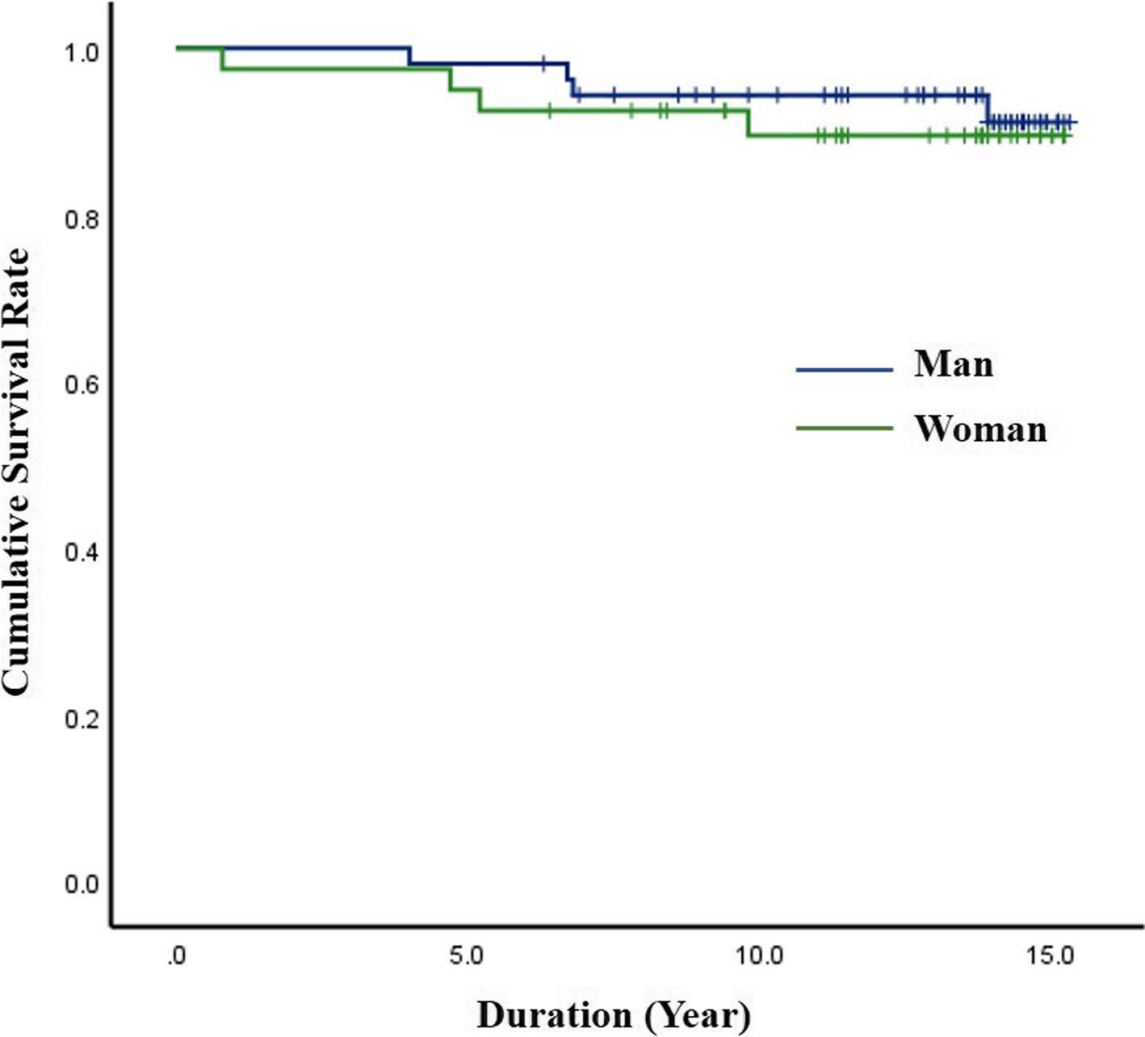
Log-rank test showed that gender did not significantly affect the reoperation rate of DIAM implantation in surgery for 1-level lumbar disc herniation (Man 8.9% vs. Woman 10.5%, *p* = 0.601)

**Fig. 4:**
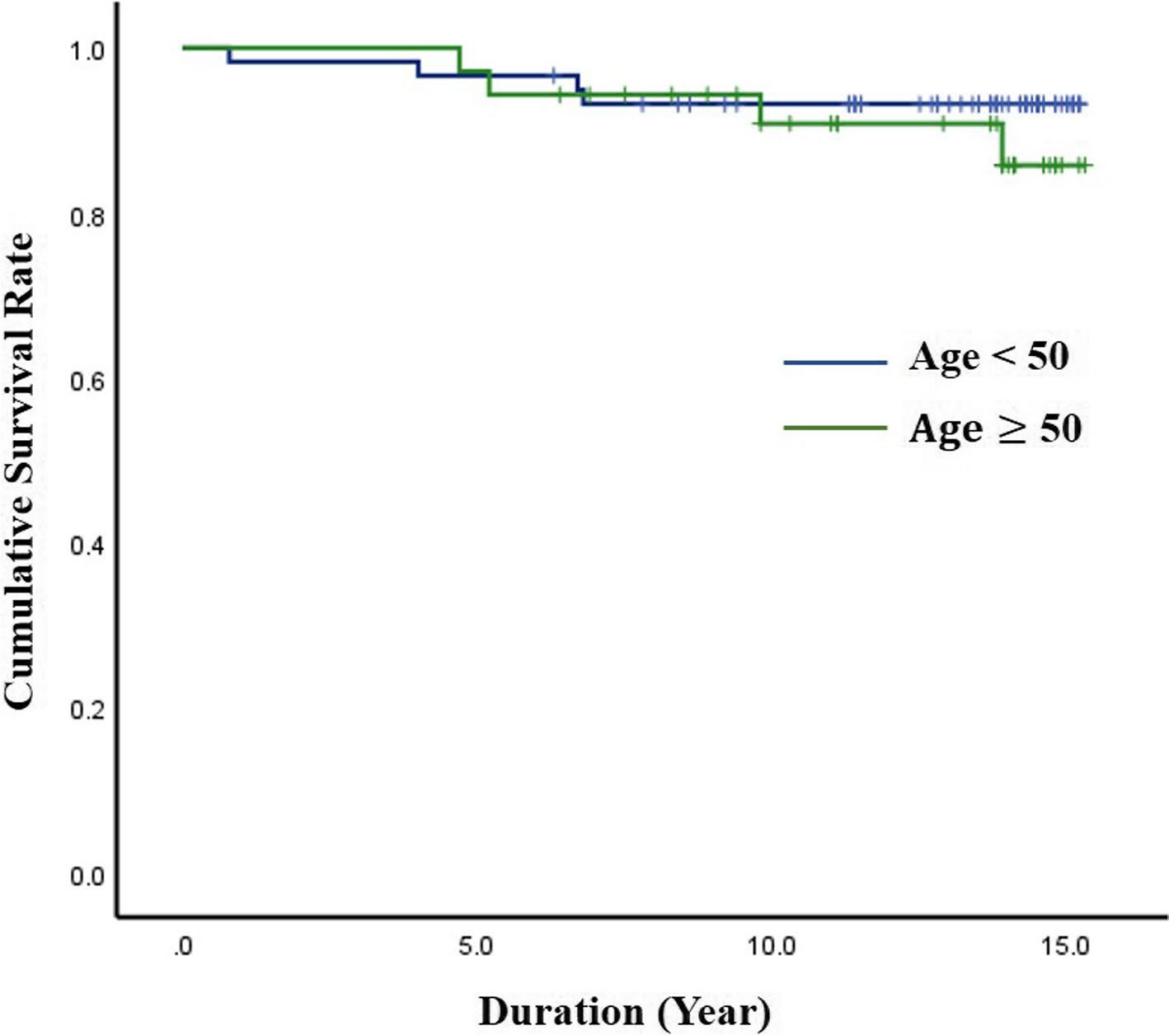
Log-rank test showed that age did not significantly affect the reoperation rate of DIAM implantation for surgery of 1-level lumbar disc herniation (Age < 50 years, 6.8% vs. Age ≥ 50 years 14.3%, *p* = 0.460)

The analysis results for reoperation rate of the DIAM implantation are summarized in Table 1. During the 15-year follow-up, 8 patients (4 men and 4 women) underwent reoperation at the DIAM implantation level, a reoperation rate of 8.5% (Fig. 1). However, no patient underwent revision surgery for adjacent segment pathologies. Reoperation rate was relatively higher in L5–6 (14.8%) compared with L4–5 (6.5%) and L3–4 (0%), but the difference was not significant (*p* = 0.415) (Fig. 2). Reoperation rate of DIAM implantation was not statistically different between < 50 and ≥ 50 years of age (6.8% vs. 11.4%, *p* = 0.465) (Fig. 3). In addition, reoperation rate of DIAM implantation was not statistically different between man and woman (7.4% vs. 10.0%, *p* = 0.719) (Fig. 4).

**Table 1:** Reoperation rate of 94 patients with 1-level discectomy and DIAM™ implantation for surgery of lumbar disc herniation

Variables	Success (%)	Failure (%)	*P-*value
Age (n = 94)			0.465^*^
< 50 years (*n* = 59)	55 (93.2%)	4 (6.8%)	
**≥** 50 years (*n* = 35)	31 (88.6%)	4 (11.4%)	
Sex (n = 94)			0.719^$^
Man (*n* = 54)	50 (92.6%)	4 (7.4%)	
Woman (*n* = 40)	36 (90.0%)	4 (10.0%)	
Location (n = 94)			0.415^≠^
L4–5 (*n* = 62)	58 (93.5%)	4 (6.5%)	
L5–6 (*n* = 27)	23 (85.2%)	4 (14.8%)	
L3–4 (n = 5)	5 (100.0%)	0 (0.0%)	

Data all represent number for each group. *P*-values are calculated by ^*^chi-square test of independence for parametric data and ^$^Fisher’s exact test for non-parametric data. *P*-value is calculated by ^≠^Fisher’s exact test for non-parametric data between L4–5 and L5-S1. Significant differences are accepted for *p*-value < 0.05

*DIAM™* Device for Intervertebral Assisted Motion

**Fig. 5:**
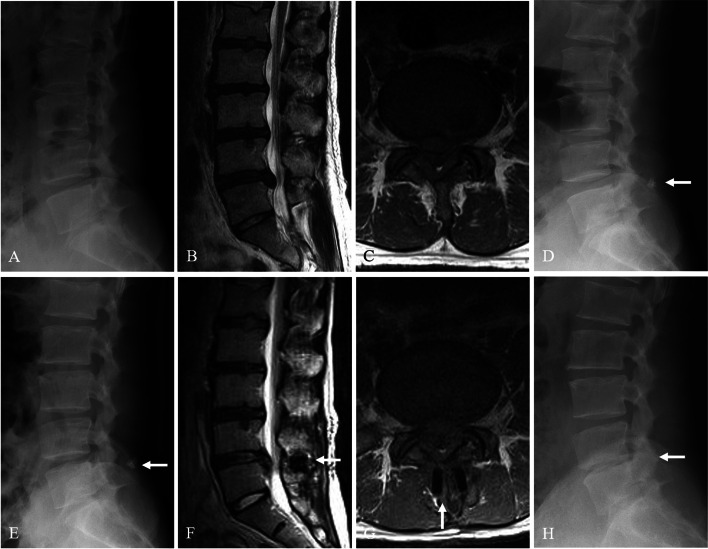
A 36-year-old woman with lumbar disc herniation (LDH) at L4–5. Lateral (**A**) radiographs showing lumbarization of S1, becoming L6. Magnetic resonance images (**B** and **C**) showing LDH at L5–6. The patient underwent discectomy and DIAM implantation at L5–6 (white arrow) (**D**). At 8 months after surgery, lateral radiograph (**E**) and magnetic resonance images (**F** and **G)** showing maintenance of DIAM (white arrows) but recurrent LDH at L5–6. At 5 years after DIAM removal and another discectomy, lateral radiograph (**H**) shows disc space narrowing at L5–6 (white arrow)

**Fig. 6:**
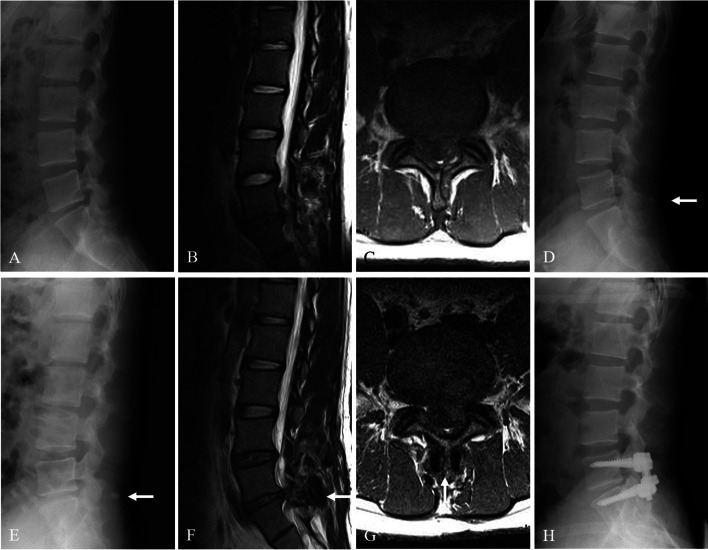
A 27-year-old man with lumbar disc herniation (LDH) at L5–6. Lateral (**A**) radiographs showing mild degenerative changes at L5–6. Magnetic resonance images (**B** and **C**) showing LDH at L5–6. The patient underwent discectomy and DIAM implantation at L5–6 (white arrow) (**D**). At 5 years after surgery, lateral radiograph (**E**) and magnetic resonance images (**F** and **G)** show maintenance of DIAM (white arrows) but recurrent LDH and stenosis at L5–6. The patient underwent DIAM removal, total laminectomy, another discectomy, and fusion at L5–6 (**H**)

The causes of reoperation were recurrence of LDH with spinal stenosis in 7 patients (4 L4–5 and 3 L5–6) and recurrence of LDH in 1 patient (1 L5–6). The mean time to reoperation was 6.5 years (range, 0.8–13.9 years): 1 patient underwent reoperation for recurrence of LDH (L5–6) at 8 months after surgery (Fig. 5); the remaining 7 patients underwent reoperation for recurrence of LDH with spinal stenosis (4 L4–5 and 3 L5–6) at mean 7.6 years (range, 5–14 years) after surgery. Regarding reoperation methods, 1 patient with LDH recurrence (L5–6) underwent removal of DIAM and another discectomy. However, 7 patients with LDH recurrence and spinal stenosis (4 L4–5 and 3 L5–6) underwent DIAM removal, extensive decompressive laminectomy, another discectomy, and posterolateral fusion with pedicular screw fixation (Fig. 6). After reoperation, satisfactory surgical outcomes were achieved in 8 patients.

**Fig. 7:**
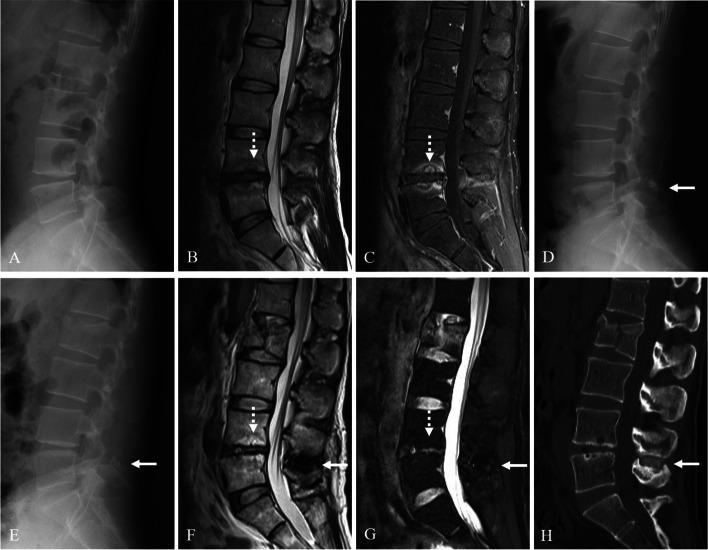
A 34-year-old woman with lumbar disc herniation (LDH) at L4–5. Lateral radiograph (**A**) and sagittal magnetic resonance images (**B** and **C**) showing degenerative changes and severe modic change (dotted white arrows) at L4–5. The patient underwent discectomy and DIAM implantation at L4–5 (white arrow) (**D**). At 11 years after discectomy and DIAM implantation at L4–5, lateral radiograph (**G**), sagittal magnetic resonance images (**F** and **G**) and computed tomogram (**H**) show well maintenance of DIAM and markedly improvement of modic changes (white arrows) at L4–5 (white arrows) without recurrence of LDH

**Fig. 8:**
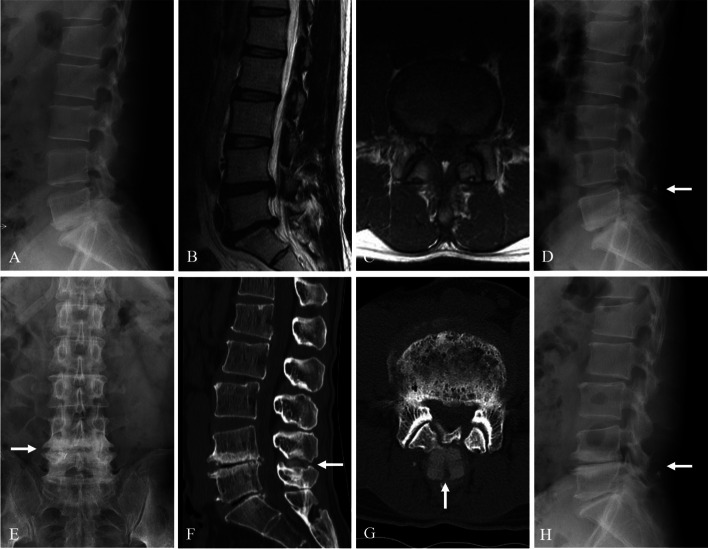
A 38-year-old man with lumbar disc herniation (LDH) at L4–5 and L5–6. Lateral (**A**) radiograph showing mild degenerative changes at L4–5 and L5–6. Magnetic resonance images (**B** and **C**) showed LDH at L4–5 and L5–6. The patient underwent discectomy and DIAM implantation at L4–5 (white arrow) (**D**). At 15 years after discectomy at L4–5 and L5–6 and DIAM implantation at L4–5, anteroposterior radiograph (**E** and **H**) and computed tomography scans (**F** and **G**) show severe degenerative changes at L4–5 and L5–6 and maintenance of DIAM at L4–5 (white arrows)

Kaplan-Meier analysis showed a cumulative survival rate of DIAM implantation of 99% at 1 year, 97% at 5 years, 93% at 10 years, and 92% at 15 years after surgery (Figs. 7 and 8). Consequently, the cumulative reoperation rate of DIAM implantation was 1% at 1 year, 3% at 5 years, 7% at 10 years, and 8% at 15 years after surgery. The log-rank test showed that location, sex, and age of DIAM implantation did not significantly affect the survival rate of DIAM™ implantation (Figs. 2, 3, 4).

Kaplan-Meier analysis showed a mean survival time of 14.5 years (95% CI, 13.97–15.07). The survival time results are summarized in Table 2. Survival time was relatively lower in L5–6 (mean, 13.5 years) compared with L4–5 (mean, 14.9 years) and L3–4 (15.0 years), but the difference was not significant (*p* = 0.191). Survival time of DIAM™ implantation was not statistically different between < 50 and ≥ 50 years of age (14.5 years vs. 14.5 years, *p* = 0.460). In addition, survival time of DIAM implantation was not statistically different between man and woman (14.7 years vs. 14.2 years, *p* = 0.601).

**Table 2:** Analysis of survival time of 94 patients with 1-level discectomy and DIAM™ implantation for surgery of lumbar disc herniation

Variables	Survival time (years)	Standard error	95% CI	*P*-value
Total (*n* = 94)	14.52	0.28	13.97–15.07	
Age (n = 94)				0.460
< 50 years	14.48	0.36	13.77–15.18	
**≥** 50 years	14.45	0.44	13.58–15.31	
Sex (n = 94)				0.601
Man	14.72	0.30	14.13–15.32	
Woman	14.17	0.51	13.17–15.12	
Location (n = 94)				0.191^≠^
L4–5	14.86	0.24	14.40–15.32	
L5–6	13.54	0.78	12.02–15.07	
L3–4	15.00	0.00		

Significant differences are accepted for *p*-value < 0.05. *P*-value is calculated by log-rank test

*DIAM™* Device for Intervertebral Assisted Motion

The multiple stepwise regression analysis with Cox regression proportional hazard model results are summarized in Table 3. Patient age (hazard ratio, 1.04; 95% CI, 0.98–1.09, *p* = 0.178), sex (hazard ratio, 2.03; 95% CI, 0.34–11.39, *p* = 0.420), and location (hazard ratio, 4.44; 95% CI, 0.79–24.91, *p* = 0.090) did not significantly affect reoperation rate of DIAM implantation in surgery for 1-level LDH. In addition, the results of univariate and multivariate regression analyses are summarized in Table 4. Univariate regression analysis showed that age (Beta, 0.54; 95% CI, 1.71; *p* = 0.196), sex (Beta, 0.63; 95% CI, 1.87; *p* = 0.503), and location (Beta, 0.26; 95% CI, 1.29; *p* = 0.181) were not significant variables associated with a higher reoperation rate (Table 4). The multivariate regression analysis showed the same results, confirming the univariate regression analysis for age (Beta, 0.54; 95% CT, 1.71; *p* = 0.473) and location (Beta, 0.26; 95% CI, 1.29; *p* = 0.115).

**Table 3:** Multiple stepwise regression analysis with Cox proportional hazard model

Variables	Hazard ratio	95% CI	*P*-value
Age	1.04	0.98–1.09	0.178
Sex	2.03	0.34–11.39	0.420
Location	4.44	0.79–24.91	0.090

Significant differences are accepted for *p*-value < 0.05

**Table 4:** Univariate and multivariate regression analysis for treatment of DIAM™ implantation

Variables	Univariate Analysis			Multivariate Analysis		
	Beta	OR (95% CI)	*P*-Value	Beta	OR (95% CI)	*P*-Value
Age	0.04	1.04 (0.98–1.11)	0.196	0.54	1.71 (0.39–7.44)	0.473
Sex	0.63	1.87 (0.30–11.66)	0.503			
Location	1.36	3.88 (0.53–28.24)	0.181	0.26	1.29 (0.30–5.60)	0.115

Significant differences are accepted for *p*-value < 0.05

In the present study, the results of a 15-year survivorship analysis of DIAM implantation were reported, which is the longest follow-up period after interspinous device surgery performed for LDH. During the 15-year follow-up, overall reoperation rate was 8.5%. All reoperations were performed for LDH recurrence at the DIAM implantation level and not for adjacent segment pathologies. The cumulative survival rate was 97% at 5 years, 93% at 10 years, and 92% at 15 years after surgery, using first reoperation due to any reason at the DIAM level or adjacent levels as the end point based on Kaplan-Meier survival analysis. The reoperation rates of 3% at 5 years, 7% at 10 years, and 8% at 15 years after surgery are satisfactory and acceptable. Therefore, DIAM implantation could be considered a useful intermediate procedure for LDH surgery.

## Discussion

Reoperation after lumbar discectomy is a significant burden for patients and spine surgeons due to less satisfactory surgical outcomes, high hospital costs, and complications compared with primary lumbar discectomy [9–11]. Therefore, prevention of reoperation is an important goal for patients and spine surgeons. Lumbar discectomy can compromise the structure of the lumbar motion segment and lead to further degeneration and excessive or abnormal motion, which result in a high recurrence rate [12–14]. Lumbar fusion is a surgical procedure to stabilize the lumbar motion segment and has been used to prevent reoperation after lumbar discectomy. However, contrary to expectations, Martin et al.’ reported that lumbar fusion did not reduce reoperation rates and suggested that an alternative other than lumbar fusion is needed [25]. As a concept of dynamic stabilization, interspinous devices were introduced in the 1990s. Since then, interspinous devices have been used widely in lumbar spine surgery as an intermediate step procedure that compensates for the shortcomings of decompression alone and fusion surgeries [26–34]. However, despite their increased popularity, the usefulness of interspinous devices remains controversial.

In previous studies, the usefulness of DIAM implantation in surgery for degenerative lumbar diseases was reported. Zhao et al. reported that the visual analog scale (VAS) for pain and the Oswestry disability index (ODI) were significantly improved in patients with lumbar vertebral instability after DIAM surgery at an average follow-up of 20.6 months [21]. Hrabálek et al. reported that all patients achieved improved clinical outcomes measured using VAS and ODI at the 3-year follow-up, and no patient treated with DIAM had any recurrence of LDH [16]. Lu et al. reported that dynamic stabilization with DIAM provided pain relief and functional improvement without compromising segmental motion at the index level or causing adjacent segment degeneration at the 3-year follow-up [17]. In addition, survivorship analysis by Sur et al. showed 8% cumulative reoperation rate 4 years postoperative in patients with lumbar spinal stenosis or LDH [19]. However, these previous studies have shortcomings such as a relatively short follow-up period and heterogenicity of the patients included in the study; thus, they do not adequately show the actual long-term effects of DIAM in surgery for LDH. Therefore, in the current study, the 15-year survivorship analysis of DIAM™ used in surgery for LDH was performed.

Current study results showed that 8.5% of the patients underwent reoperation at the DIAM implantation level during the 15-year follow-up. The mean time to reoperation was 6.5 years (range, 0.8–13.9 years). Although only 1 patient underwent reoperation within 1 year after surgery, the remaining 7 patients underwent reoperation at least 5 years after surgery. Because most reoperations are performed within 1 year after surgery in LDH patients who had lumbar discectomy only, these findings are encouraging for patients and spine surgeons. Discectomy alone surgery for lumbar disc herniation results in early favorable outcome. However, the outcome usually deteriorates over time due to destabilizing effect of discectomy to motion segment, such as loss of disc height and facet joint overloading. On the contrary, discectomy surgery with DIAM stabilize the motion segment by restriction of segmental motion and offset of facet joint overloading, which can reduce the recurrence of lumbar disc herniation. We speculate that additional DIAM implantation can effectively prevent LDH recurrence of LDH by providing dynamic stabilization to the lumbar discectomy segment, and this biomechanical effect lasts for 15 years. Therefore, DIAM implantation can significantly decrease the reoperation rate compared with lumbar discectomy only. Another important finding is that none of the patients underwent reoperation for adjacent segment pathologies. Compared with lumbar fusion that frequently causes adjacent segment pathologies, this finding indicates that DIAM implantation does not increase abnormal biomechanical stress to the adjacent segments.

Log-rank test and univariate and multivariate regression analyses showed that age, sex, and location of DIAM implantation did not significantly affect reoperation rate for 1-level LDH surgery. Reoperation rate was relatively higher in L5–6 compared with L4–5 and L3–4, but the difference was not significant (*p* = 0.415). In addition, survival time was not significantly different by age, sex, or location. However, in a previous study by Sur et al., the 4-year survivorship analysis of DIAM implantation in surgery for lumbar spinal stenosis and LDH showed the reoperation rate to be significantly higher in L5–6 compared with L4–5 (*p* < 0.01) [19]. Survival time also was significantly lower in L5–6 compared with L4–5 (*p* < 0.01). The conflicting result between our study and the study by Sur et al. is possibly due to the difference in pathology of lumbar spine diseases. In the present study, only 1-level LDH was targeted, whereas in the study by Sur et al., two types of degenerative lumbar diseases were targeted, including LDH and spinal stenosis, and included 2-level surgery [19]. Even when considering these differences, the decision to DIAM implantation when performing L5–6 LDH surgery should be carefully made [26–35]. In a previous study, the recurrence rate after lumbar discectomy only was reportedly high in patients < 50 years of age [5]. Apparently, high physical activity at a young age influences the recurrence of LDH. However, the results of our study are different from previous studies in which only lumbar discectomy was performed. Although statistically significant difference was not observed, the reoperation rate after discectomy and DIAM implantation in surgery for 1-level LDH was slightly higher in patients ≥50 years of age compared with < 50 years of age. Therefore, more in-depth follow-up studies are needed in which the effect of age on reoperation after DIAM surgery are investigated.

There are some limitations in the study. First, we did not include control group (lumbar disc herniation without interspinous device) in the study, which affects the conclusion of the study. Second, even though statistically not significant, survival rate and time were relatively lower at L5–6 compared to L4–5. So, careful consideration should be given the use of interspinous device to L5–6 level.

## Conclusion

The present study showed that DIAM implantation significantly decreased reoperation rate for 1-level LDH in 15-year survivorship analysis. We suggest that DIAM implantation could be considered a useful intermediate step procedure in surgery for 1-level LDH. To the best of our knowledge, this is the longest follow-up study in which surgical outcomes of interspinous device surgery were reported to date.

## Data Availability

The datasets used and/or analyzed during the current study are available from the corresponding author on reasonable request.

## Abbreviations

DIAM: Device for Intervertebral Assisted Motion
LDH: Lumbar disc herniation
VAS: Visual analog scale
ODI: Oswestry Disability Index
CI: Confidential interval
